# Case Report: Two children with factor XII deficiency caused by novel *F12* compound heterozygous variants

**DOI:** 10.3389/fped.2025.1555426

**Published:** 2025-07-02

**Authors:** Rui-Xue Ma, Hai-Yan Li, Yi-Hang Zhang, Xue-Min Zhang, Yan-Juan Chen, Yi-Lin Dai, Gui-Xian Li, Wen-Hai Luo, Jie Zhang, Yun-Fen Tian

**Affiliations:** ^1^ College of Medicine, Kunming University of Science and Technology, Kunming, Yunnan, China; ^2^ Department of Pediatrics, The First People's Hospital of Yunnan Province, The Affiliated Hospital of Kunming University of Science and Technology, Kunming, Yunnan, China; ^3^ Department of Pediatrics, The First People's Hospital of Anning City, Anning, Yunnan, China; ^4^ West China School of Medicine, West China Hospital, Sichuan University, Chengdu, Sichuan, China; ^5^ Department of Medical Genetics, The First People's Hospital of Yunnan Province, The Affiliated Hospital of Kunming University of Science and Technology, Kunming, Yunnan, China

**Keywords:** *F12*, hereditary coagulation factor XII deficiency, prolonged APTT, children, gene mutation, novel mutation

## Abstract

**Background:**

Factor XII (FXII) deficiency (OMIM 234000) is a rare hereditary coagulation disorder caused by pathogenic variants within the *F12* gene. It causes prolonged activated partial thromboplastin time without bleeding diathesis. Most patients have no obvious clinical symptoms, so the disease is difficult to be detected.

**Case presentation:**

Here, we reported two pediatric cases with FXII deficiency from Kunming, China. Patient 1 was a 10-year-old girl who was hospitalized with a fever and cough for one week and diagnosed with pneumonia. Auxiliary coagulation function examination suggested that the activated partial thrombin time (APTT) was significantly prolonged, while both the coagulation factor XII activity (FXII:C) and coagulation factor XII antigen (FXII:Ag) were decreased. Whole exome sequencing (WES) revealed this patient carries *F12* compound heterozygous variants with NM_000505.4:c.509G>A (p.Cys170Tyr) and NM_000505.4:c.800+1G>C. Patient 2 was a newborn boy with prolonged coagulation of the umbilical cord and difficult hemostasis after birth. A prolonged APTT and a decreased ratio of FXII:C were observed. WES revealed this patient carries *F12* compound heterozygous variants with NM_000505.4:c.583del (p.His195Thrfs*56) and NM_000505.4:c.805C>T (p.Pro269Ser). *In vivo* RT-PCR assays demonstrated c.800+1G>C intron mutation resulted to a 166-bp deletion (exon 8 skipping) for patient 1. Bioinformatics analysis confirmed the pathogenicity of all four variants.

**Conclusions:**

We presented two pediatric cases with FXII deficiency caused by novel *F12* compound heterozygous variants. Pediatricians should raise awareness of this rare and underdiagnosed disorder and improve diagnostic and intervention strategies.

## Introduction

FXII deficiency (OMIM 234000) is a rare hereditary coagulation disorder caused by pathogenic variants within the *F12* gene, which is characterized by prolonged activated partial thromboplastin time (APTT) without excessive bleeding (1). The *F12* gene is located on chromosome 5q33-qter and encodes FXII, a serine protease mainly synthesized in the liver, which formerly known as the Hageman factor (2). FXII plays a critical role in activation of the intrinsic coagulation pathway, the kallikrein-kinin system, fibrinolysis, and complement system (3, 4). FXII deficiency is normally asymptomatic, and the majority of the cases are diagnosed through preoperative coag­ulation screening or during a health check. In this report, we present two pediatric cases of FXII deficiency caused by novel compound heterozygous variants on *F12* from Yunnan, China.

## Case presentation

### Medical history

Patient 1 was a 10-year-old girl. She was admitted to our hospital with the symptom of cough and fever for one week. She was diagnosed as mycoplasma infection with a positive of mycoplasma antibodies, and was treated with cefuroxime infusion and azithromycin tablets for 3 days in a local hospital. However, the symptoms did not improve, and a chest CT scan indicated solid right lung and pleural effusion. Then she was admitted to our department for further treatment. She was the first child of a healthy non-consanguineous couple, no previous history of bleeding or liver disease, no anticoagulant medication was reported, and her 4-year-old sister was in good health. On admission, physical examination showed a normal weight of 35 kg, HR 100/min, RR 21/min, BP 98/62 mmHg, and body temperature 39℃. The pharynx was congested, and the tonsils were not enlarged. The right lung was turbid on percussion, the breath sounds were slightly low, and a few fine moist rales could be heard in the right lung. No clinical signs of deep vein thrombosis. Her other systemic examinations were unremarkable.

Patient 2 was a newborn boy. He was born full-term by vaginal delivery with a birth weight of 2.9 kg. His family history was unremarkable for hematologic disorders. General physical examination showed a HR 155/minute, RR 48/min, and body temperature 38.3℃. After birth, the coagulation at the end of the umbilical cord was prolonged, and hemostasis at the puncture site was difficult.

Blood routine testing and coagulation assays were performed in both patients and their close relatives. As shown in Table 1, the results revealed a significant prolonged APTT and markedly decreased levels of coagulation FXII activity (FXII: C) in both patients. FXII antigen (FXII: Ag) was significantly decreased in patient 1, and PT was prolonged in patient 2. The results of their close relatives showed a slight prolonged APPT and PT, but the FXII: C and FXII antigen were normal (Table 1).

**Table 1:** Laboratory characteristics of patients and their family members.

Subject	WBC (10^9^/L)	Hb (g/L)	Plt (10^9^/L)	APTT^a^ (s)	PT^b^ (s)	FXII:C^c^ (%)	FXII:Ag^d^ (%)
Patient 1	7.3	124	390	133.1	13.2	2.00	3.90
Father of patient 1	9.8	135	264	35.2	12.0	80.00	75.00
Mother of patient 1	11.2	126	185	40.5	13.8	95.00	110.00
Sister of patient 1	10.5	143	210	39.4	22.8	113.00	96.00
Patient 2	12.7	195	80	121.3	32.7	0.80	102.00
Father of patient 2	8.5	130	232	43.3	14.2	83.00	98.00
Mother of patient 2	11.6	115	180	38.5	10.5	95.00	76.00

^a^ APTT normal value 27.0 s∼41.0 s.

^b^ PT normal value 12.5 s∼14.5 s.

^c^ FXII:C (%) normal value 72%∼113%.

^d^ FXII:Ag (%) Normal value 72%∼113%.

### WES and Sanger sequencing

To further confirmed the diagnosis of FXII deficiency, WES by using genomic DNA extracted from peripheral blood was performed in both patients. Sequence alignment was performed using BAM-mem. The initial analysis and variant detection of the BAM file as well as variant filtering were carried out using GATK4. Using ANNOVAR, public crowd frequency database and disease database to proceeded Variant annotation. The analysis and interpretation of variations mainly focused on the variations related to the phenotype of the probands.

**Figure 1:**
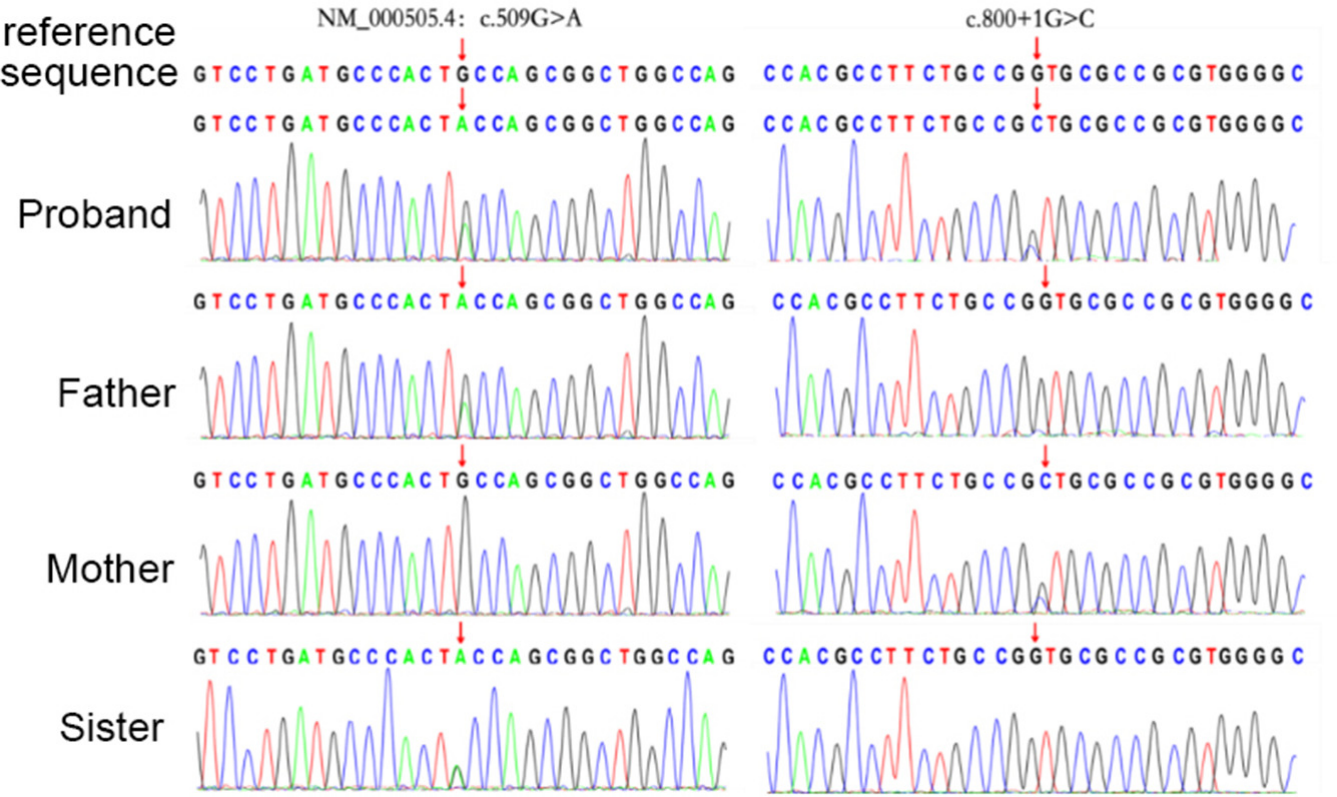
Sanger sequencing confirmed variants in the *F12* gene in the patient 1, her parents, and her sister.

**Figure 2:**
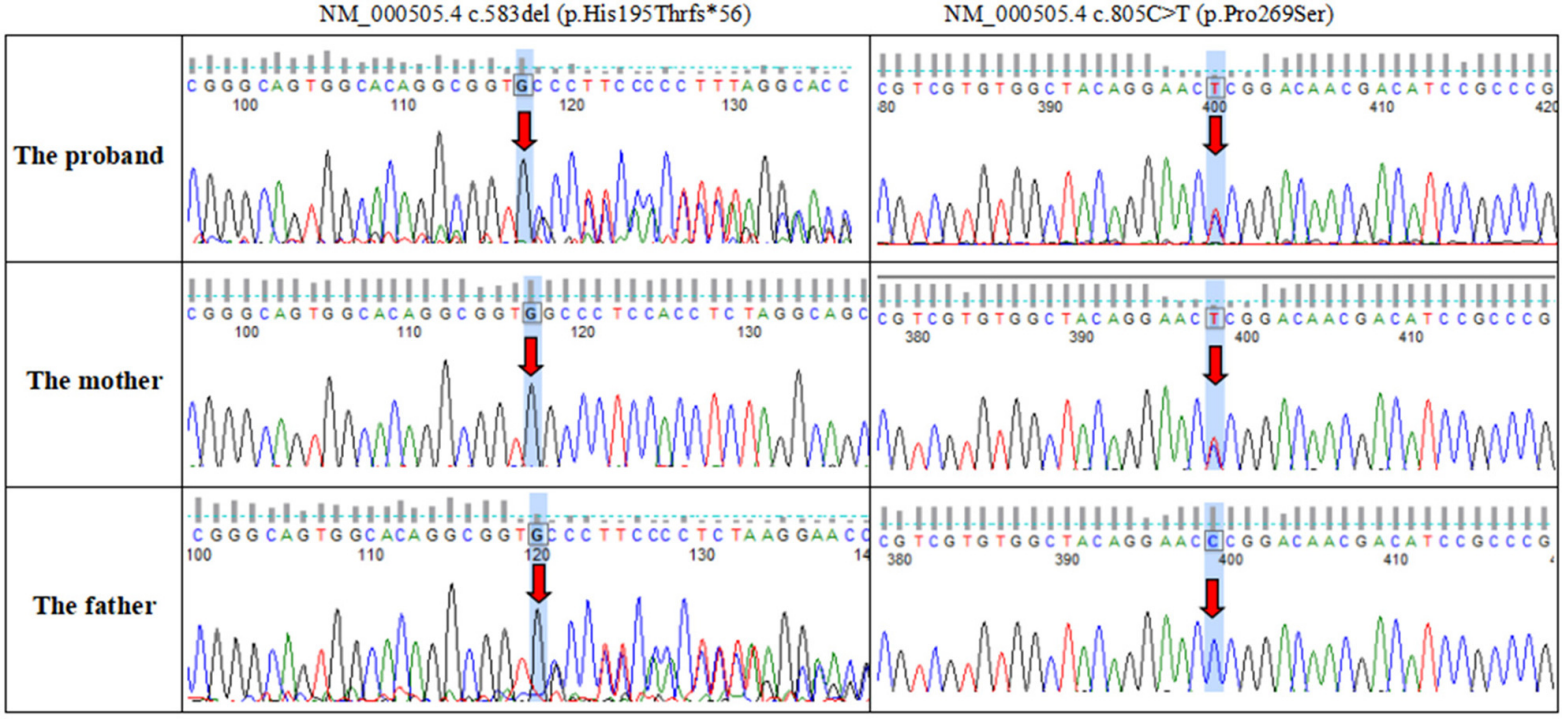
Sanger sequencing confirmed variants in the *F12* gene in the patient 2 and his parents.

Novel compound heterozygous variants with NM_000505.4:c.800+1G>C and NM_000505.4:c.509G>A (p.Cys170Tyr) were identified in the patient 1. Further genotyping of the family members by Sanger sequencing showed that NM_000505.4:c.800+1G>C was maternally derived and NM_000505.4:c.509G>A was paternally derived. The unaffected sister carried a heterozygous variant with NM_000505.4:c.509G>A (Figure 1). Patient 2 had compound heterozygous mutations of *F12*, NM_000505.4:c.583del (p.His195Thrfs*56) and NM_000505.4:c.805C>T (p.Pro269Ser). Sanger sequencing of the patient's parents confirmed that NM_000505.4:c.583del was paternally inherited, NM_000505.4:c.805C>T was maternally inherited (Figure 2). Thus, the diagnosis of FXII deficiency was made in both patients.

*F12* gene were amplified using the following primers: for c.509G>A mutation, F 5'CTTGGGGAGGGAACGCAGTGA3', R 5'ATAGGTTGCTGGATACTCGGAGAC3'; for c.800+1G>C and c.805C>T, F 5'GCGGATGTCGTTGTCCGGGT3’, R 5'CAGGGGTGGCTCACTGCGTT3’. for c.583del, F 5'CCAGAAAGGTGAGGAGATGTG3', R 5'CCTTGGTGTCTGAGGAGAAAG3'.

### Bioinformatics analysis of gene variant

The pathogenicity of point mutation was evaluated using the HumDiv-trained model in Polyphen-2 and SIFT (http://sift.jcvi.org). The pathogenicity of the intron mutation was assessed using the MaxEntScan, dbscSNV, spliceAI. For point deletion mutation, the effect of the mutation on resulting peptide was evaluated using mutalyzer (https://mutalyzer.nl).

Mutation NM_000505.4:c.509G>A and NM_000505.4:c.805C>T were predicted as “deleterious” by bioinformatics sofware. Mutation NM_000505.4:c.800+1G>C was predicted as “deleterious” for aberrant splicing. For mutation c.583del, the nucleotide frameshift would result in a shortened protein according to mutalyzer software.

Mutation NM_000505.4:c.583del lead to a completely F12 protein functional lose, presumably leading to obvious FXII:C deficiency (0.80%) for Patient 2 compared to patient 1 (2.0%).

### RT-PCR assay *in vivo*

**Figure 3:**
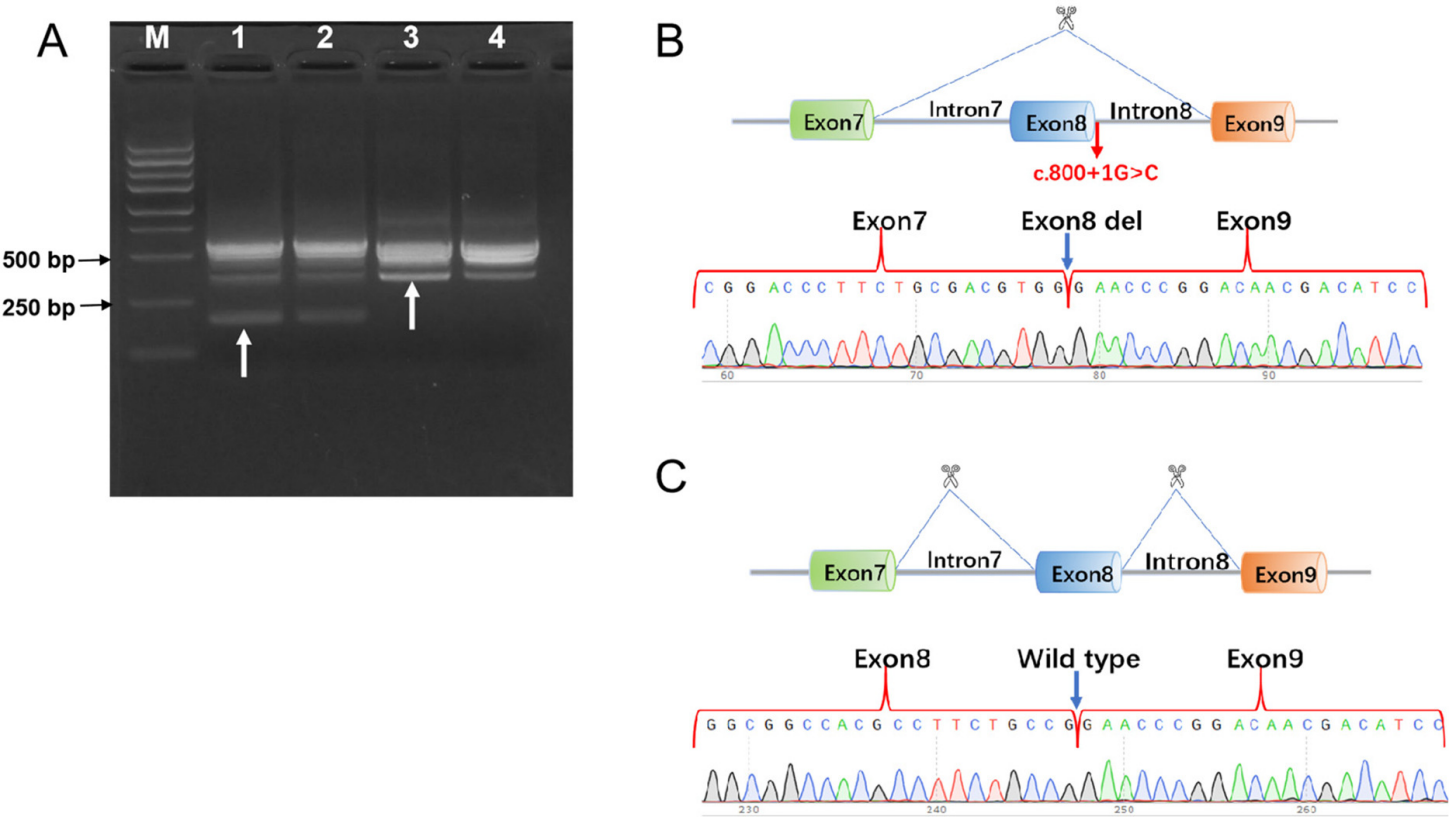
*In vivo* RT-PCR validation of F12 exon 8 deletion in the proband. **(A)** Nucleic acid electrophoresis results: Lane M: DNA ladder (Marker). Lane 1 and Lane 2: The proband and mother exhibited a 204 bp and a 370 bp fragment. Lane 3 and Lane 4: The father and negative control exhibited a 370 bp fragment. **(B)** Sanger sequencing chromatogram alignment, confirming that the c.800+1G>C mutation resulted in a 166-bp deletion causing exon 8 skipping. **(C)** Schematic view of wild-type exon 8 sequencing.

In addition, *in vivo* RT-PCR was performed directly on blood samples from patient 1 and family members to validate the *F12* splicing variant. *F12* gene segment encompassing exon 7 to exon 9 were amplified using nested PCR. Primers for the first PCR amplification was E6-F 5'AGTGCAAGGGTCCTGATGCC3' and E10-R 5'AGACAGACTCTTGCGGAGCC3'. Primers for the second PCR amplification was E7-F 5'CCTGCCGCACCAACCCGT3' and E9-R 5'TGGGTTGGGGTCTGGCACT3'. Nucleic acid electrophoresis of the PCR products indicated that the proband and his mother carried a heterozygous variant, displaying two bands: a shorter 204 bp band corresponding to exon 8 deletion and a 370 bp band representing the normal allele. The proband's father and negative controls showed no variant, with a single 370 bp fragment (Figure 3A).

Sanger sequencing analysis of the proband and mother revealed one sequence with exon 8 deletion and another with normal sequencing chromatograms. In contrast, the proband's father and negative control exhibited wild-type exon 8 sequencing chromatograms (Figures 3B,C).

### Symptomatic treatment and follow-up

Patient 1 had no symptoms of bleeding or embolism, so no special treatment was made for FXII deficiency. After anti-inflammatory treatment, her body temperature was normalized on the third day, the cough symptoms improved, and the coagulation function was rechecked before discharge (APTT = 149.8 s). The child was discharged from the hospital and has been followed up regularly in the outpatient clinic without any specific abnormal clinical manifestations. Patient 2 was injected with plasma to supplement coagulation factors during hospitalization, APTT was normal after re-examination (APTT = 35 s), and his condition was stable after discharge. His APTT was still prolonged during the follow up, while he has no bleeding or thrombosis occurred so far.

## Discussion

Hereditary FXII deficiency is a rare autosomal recessive condition that results in prolonged activated partial thromboplastin time due to reduced activity of coagulation factors XII (1). Inherited FXII deficiency often manifests as prolonged clotting time, but spontaneous bleeding is less common and patients are usually asymptomatic, as also demonstrated in FXII knockout (FXII^−^/^−^) mice (5). The extrinsic coagulation pathway is the main pathway of coagulation in the body, and its activation form a complex that also activates coagulation FXI mediated endogenous coagulation leading to clotting, which explains the less incidence of spontaneous bleeding in patients with FXII deficiency (6). No bleeding symptoms occurred in our reported FXII deficiency cases after birth, which is consistent with the previous findings. In addition, some studies have shown that FXII has an essential role in pathological thrombus formation (1, 7). So the defect of the *F12* gene leads to the deficiency of FXII, thereby inhibiting the enzymatic cascade waterfall activation reaction of the endogenous coagulation pathway, which can prevent the production of massive fibrin and thrombus and thus inhibit the formation of occlusive thrombosis (8–12). Hu Can (13) retrospectively analyzed 29 patients with hereditary FXII deficiency whose parents were without the disease and denied any family history or bleeding disorders, four of whom had bleeding symptoms, and one presented with menorrhagia. Normalization of coagulation APTT after transfusion of fresh frozen plasma (FFP) in a child with a compound heterozygous mutation causing hereditary FXII deficiency. In our case, patient 1 is a girl and her menstruation has started and without menorrhagia.

Human blood coagulation FXII is coded by the *F12* gene that is located on the chromosomal band 5q33-qter. FXII is primarily produced by hepatocytes and secreted into circulating plasma as a mature protein with a concentration of 30∼35 μg/ml (14). FXII deficiency can be caused by the lack of the corresponding gene, and the polymorphism of the *F12* gene also affects FXII antigen activity and content (2, 15). The test was not performed on the two patients because of the unavailability of the FXII concentration test in Yunnan province. According to the different contents of FXII: Ag and FXII: C, hereditary FXII deficiency could be divided into three phenotypes: cross-reacting material negative (CRM-), FXII: C level decreased and FXII: Ag level cannot be detected; cross-reacting material positive (CRM+), FXII: C level decreased but FXII: Ag level was normal; cross-reacting material reduction (CRMred), both FXII: C level and FXII: Ag decreased (16). In patient 1, APTT was significantly prolonged, and FXII: C and FXII: Ag were simultaneously reduced to almost zero levels, which belong to CRM- type. While APTT of patient 2 was significantly prolonged, the level of FXII: C was extremely reduced, but the level of FXII: Ag was normal, which belong to CRM+ type.

**Figure 4:**
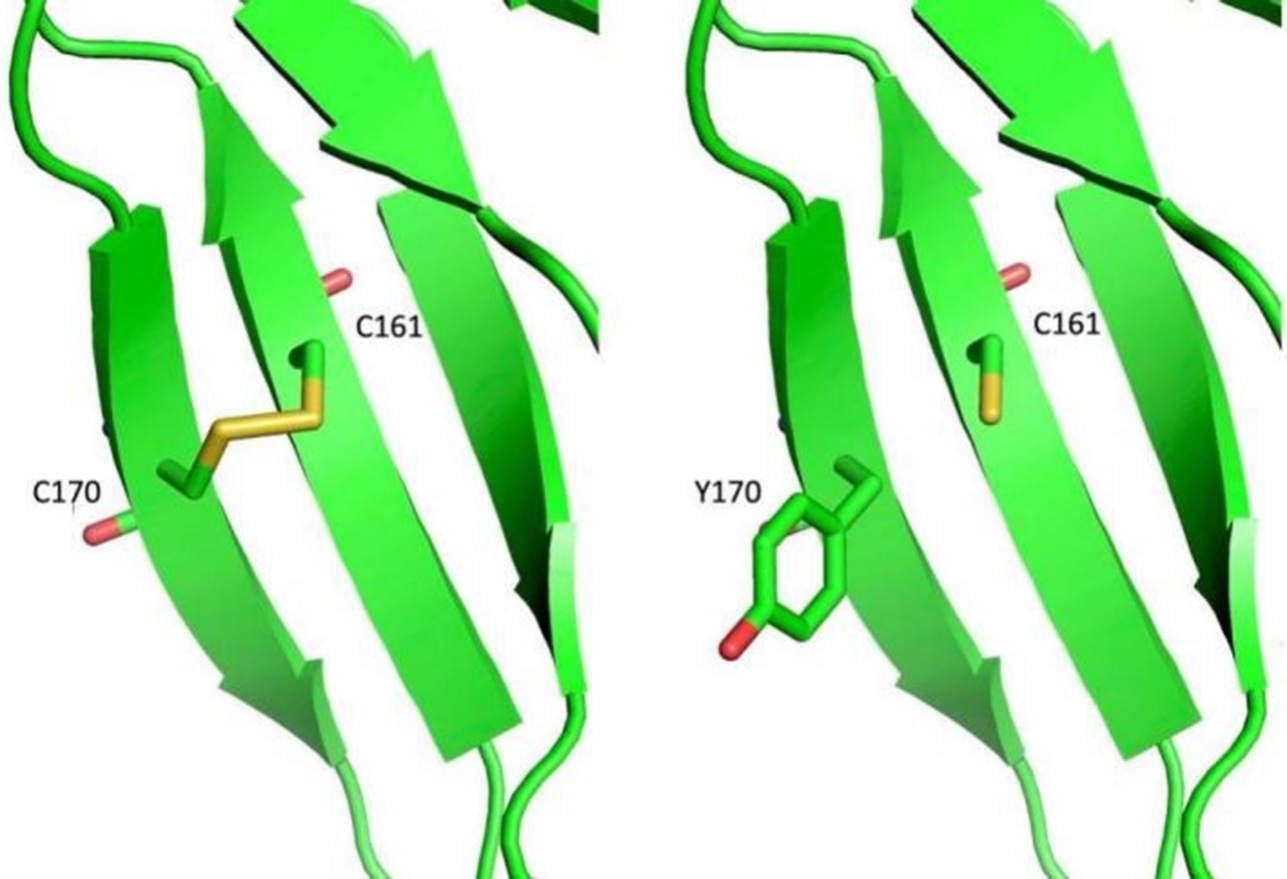
Simulation analysis of the C170Y variant in the F12 protein chain.

We identified three novel mutations in these two cases. For patient 1, NM_000505.4:c.509G>A (p.Cys170Tyr) was paternal, and NM_000505.4:c.800+1C>G was maternal. Both of these two mutations were absent in Exome Sequencing Project, 1000 Genomes or Exome Aggregation Consortium. Neither the Clivnar nor the HGMD databases have relevant records or reports of mutation. The site Cys170 is located on the Fibronectin type-IFN1structural domain of FXII and is involved in binding to fibronectin together with epidermal growth factor (17). Analysis by protein chain modeling showed that Cys170 and Cys161 are an adjacent pair of cysteines forming a disulfide bond between them, the latter being disrupted by the missense mutation Cys170Tyr, whose functional effects on the FN1 structural domain and FXII are subject to further basic studies (Figure 4). Variant NM_000505.4:c.800+1G>C occurs at the classical splicing site and is predicted to result in aberrant splicing of exon 8 skipping which would significantly alter the structure and function of the FXII protein. And this exon 8 skipping had been confirmed by *in vivo* RT-PCR performed on blood samples from this family.

For patient 2, NM_000505.4:c.583del (p.His195Thrfs*56) is paternal, and NM_000505.4:c.805C>T (p.Pro269Ser) is maternal. Both of these two mutations were absent in Exome Sequencing Project, 1000 Genomes or Exome Aggregation Consortium. Neither the Clinvar nor the HGMD databases have relevant records or reports of mutation NM_000505.4:c.805C>T. Mutation NM_000505.4:c.583del has been record as disease causing mutation (18). Mutation NM_000505.4:c.583del lead to complete loss of normal protein function through nonsense-mediated mRNA degradation or premature termination of the coding amino acid sequence, presumably leading to obvious FXII:C deficiency (0.80%) for Patient 2 compared to patient 1 (2.0%).

None of the 2 cases had active bleeding after follow-up. Therefore, it suggests that clinicians can dynamically observe and further diagnose patients with abnormal coagulation function, such as those without obvious bleeding symptoms and thrombosis, and avoid unnecessary use of blood products.

## Conclusion

In summary, we presented two pediatric cases with FXII deficiency caused by novel *F12* compound heterozygous variants. Pediatricians should raise awareness of this rare and underdiagnosed disorder and improve diagnostic and intervention strategies.

## Data Availability

The datasets presented in this study can be found in online repositories. The names of the repository/repositories and accession number(s) can be found in the article/Supplementary Material.
